# Supplementary material for: Geographic distributions and patterns of co-occurrence among black-bellied and shovel-nosed salamanders (Desmognathus spp.) in the Great Smoky Mountains National Park
Source: PeerJ. 2025 Sep 25;13:e20110. doi: 10.7717/peerj.20110 (PMC12476860; doi:10.7717/peerj.20110)
Supplement: Supplemental Information 1 — This document details the sampling procedure, including IACUC, ethical considerations, and permitting information, and provides additional information on the DNA methods used in this study. [file peerj-13-20110-s001.docx]

**Geographic distributions and patterns of co-occurrence among black-bellied and shovel-nosed salamanders (*Desmognathus* spp.) in the Great Smoky Mountains National Park**

Aidan Shaw, Rebecca T. Chastain, Benjamin M. Fitzpatrick

**Justification for sampling protocols and adherence to legal and ethical standards**

Tissue sampling was carried out in compliance with the United States Animal Welfare Act [7 U.S.C. 2131 et seq.] and according to University of Tennessee Institutional Animal Care and Use Committee (IACUC) protocol 2710 and National Park Scientific Research Collection Permit GRSM-2023-SCI-2209.

Since a major goal of this study was to understand the geographic distributions of newly described cryptic species, we could not replace live animal sampling with alternative study methods without compromising our ability to describe distributions and patterns of co-occurrence. As such, we sought to refine sampling techniques to minimize harm to animals by using the clean pinch method of tissue collection. The clean pinch method is applied with the animal in a clean zipper-lock bag to avoid skin damage and water loss. We apply a firm, gradual pinch through the bag to the distal part of the tail (0.5 to 1.0 cm) with fingers or a smooth-edged instrument until the animal autotomizes the tail tip. This is done rapidly (under 1s) and without tearing the bag. Once the salamander has autotomized the tail tip, it is released as quickly possible, and the tissue sample is transferred to a labeled sample vial for transport.

Rainey et al. (2024) compared toe clips, buccal swabs, and skin swabs as DNA samples for a small species of frog. They found that DNA yield and quality was significantly better with toe clips, and that skin swabs were particularly poor sources of animal DNA. For genome scale DNA technologies, large amounts of high-purity DNA are critical to the success of the laboratory methods, and poor samples are often wasted (meaning the animal was handled for no benefit whatsoever). Moreover, buccal swabs require significantly more coercive handling than tail samples, with non-trivial risk of damage to the teeth and tongue, which are critical for effective feeding.

For salamanders, tail samples obtained by the clean pinch method are far less damaging to the animals than toe clips but provide the same sample quality. Polich et al. (2013) and Segev et al. (2015) found no evidence that tail-clipping affected growth and survival of larval salamanders, which is noteworthy considering the importance of the tail in swimming. Adult salamanders autotomize sections of their tails in response to localized pinching by physically releasing the nearest proximal myoseptum. When autotomy occurs on these natural cleavage planes, there is minimal bleeding, and the wound is rapidly sealed by intrinsic physiological mechanisms. Dinsmore (1977) found that regeneration after natural autotomy was faster and less likely to scar muscle tissue than after surgical tail clipping.

Finally, in accordance with our goal of describing distributions and patterns of co-occurrence, we opted to sample up to 50 individuals per locality to maximize our chances of detecting rare species in a system where 3-4 genetically distinct, yet morphologically ambiguous, species could co-occur. Specifically, since we are unable to confidently differentiate between Pisgah and Nantahala black-bellied salamanders (*D. mavrokoilius* and *D. gvnigeuswotli*) and our sampling protocol was designed with future population genetics study in mind, we want to ensure that we have sufficient samples of each species to measure gene flow within and between local streams. A sample size of N=50 gives a <1% chance of missing a cryptic species that makes up 10% of the population.

**Molecular Methods and Sequence Processing**

To efficiently assign mtDNA clade for each sample we amplified approximately 500 bp of cytochrome B to compare with the existing dataset generated by Jackson (2005). We extracted DNA from tail tissues using the DNeasy Blood and Tissue kit (Qiagen) or through salt precipitation (Sambrook & Russell, 2001). All samples beginning with “GSM23” were extracted via salt precipitation. All samples beginning “GSM24” and B24D01 were extracted using the kit. Tissues extracted using both methods were incubated in lysis buffer and proteinase K overnight. DNA extraction yields quantified by Nanodrop (ThermoFisher) varied by extraction method (p(t_df=22.8_) < 0.001). The median DNA concentration for the salt precipitation extractions was 22.7 ng/ul (SE: 2.8) and that for the DNeasy extraction kit was 212.3 ng/ul (SE: 38.2).

We performed PCR using the primers cytbegin (5`-GCCCACACTTTACGAAAAAC-3`) and cytb511R (5`-GCTTTATCTACTGAAAACC-3`) created by Jackson (2005). We performed 25ul reactions using 2x DreamTaq Mastermix (ThermoFisher), 1ul of each forward and reverse primer, 1.5 ul template DNA, and 9 ul nuclease-free water. The reactions were incubated on an Eppendorf Mastercycler using the following protocol: 96˚C for 3 min; 35 cycles of 94˚C for 1 min, 48˚C for 1 min, and 72˚C for 1min; 72˚C for 7 min. We confirmed amplification using 1.5% agarose gels. We then purified PCR products using ExoSAP-IT according to the manufacturer’s (ThermoFisher) instructions. Samples were then sequenced using Eurofins Genomics’ PlateSeq service.

We aligned and visualized sequences using UGENE (Unipro). We removed low-quality regions at either end of the alignment. The sequences used in the phylogenetic analysis and our genetic distance analysis are available in GenBank. Upon aligning our 270 sequences to 373 existing sequences from Jackson (2005), we noted a run of repeating A’s and T’s (beginning at position 184 in sequences on GenBank) that were of variable length throughout our dataset. These apparent length variations caused frameshifts in many of our sequences, introducing internal stop codons. Visual inspection of chromatograms instilled doubt as to whether we would be able to accurately infer the length of that region for any of our sequences. We suspect that this length variation was a result of sequencing error. Stutter peaks due to homopolymer repeats are a known challenge in Sanger sequencing (Al-Shuhaib & Hashim, 2023). To avoid introducing any unnecessary assumptions and to make alignment easier, we replaced these runs with 10 N’s, signifying missing data, which, while not biologically meaningful, allowed the sequences to be aligned to existing data and maintained the reading frame, eliminating internal stop codons.

Our resulting alignment consisted of our 270 sequences and Jackson’s (2005) 373 sequences, which included some representatives from other *Desmognathus spp.* and one from *Phaeognathus hubrichti*. We then used BEAST and MrBayes to estimate gene trees for our sample. In BEAST, we specified *P. hubrichti* as the outgroup (Pyron et al., 2020). We used the built-in Yang96 site partitioning model which allows for independent substitution rates and base frequencies across codon positions and uses a GTR-G substitution model. Our BEAST run used a MCMC chain of 40M generations. Our MrBayes run also used a site partitioning model based on the one used by Jackson (2005) and a MCMC chain of 40M generations.

The gene tree that we present is the Maximum Clade Credibility tree produced by the TreeAnnotator software from the output of BEAST. The tree figures were created using *ggtree* in R (v3.12.0, Yu, 2018).

We created the haplotype network presented in figure 5 as a visual aid to accompany the genetic divergence analyses performed on Pisgah clade populations. The haplotype network was created from the 91 sequences used in the genetic distance analysis which represent samples from four populations (Onion Bed Branch, Rough Fork, Pretty Hollow, and Cosby) for which we had at least 10 Pisgah clade individuals. The haplotypes were estimated using the function *pegas::haplotypes*, the sequence divergence was estimated using *ape::dist.dna*, and the maximum parsimony haplotype network was estimated using *pegas::haploNet* (Paradis, 2010, 2012).

**References**

Al-Shuhaib MBS, Hashim HO. 2023. Mastering DNA chromatogram analysis in Sanger sequencing for reliable clinical analysis. *Journal of Genetic Engineering and Biotechnology* 21:115. DOI: [10.1186/s43141-023-00587-6](https://doi.org/10.1186/s43141-023-00587-6).

Beaupre SJ, Jacobson ER, Lillywhite HB, Zamudio K. 2004. Guidelines for Use of Live Amphibians and Reptiles in Field and Laboratory Research, 2nd ed. American Society of Ichthyologists and Herpetologists.

Dinsmore CE. 1977. Tail regeneration in the plethodontid salamander, Plethodon cinereus: induced autotomy versus surgical amputation. *J Exp Zool*. 199:163–75. DOI: 10.1002/jez.1401990202.

Jackson N. 2005. Phylogenetic History, Morphological Parallelism, and Speciation in a Complex of Appalachian Salamanders (Genus Desmognathus). *Theses and Dissertations*.

Paradis E. 2010. pegas: an R package for population genetics with an integrated–modular approach. Bioinformatics 26: 419–420. doi:10.1093/bioinformatics/btp696.

Paradis E. 2012. *Analysis of Phylogenetics and Evolution with R (Second Edition).* New York: Springer.

Polich RL, Searcy CA, Shaffer HB. 2013. Effects of tail-clipping on survivorship and growth of larval salamanders. *J Wildl Manage* 77:1420–1425. DOI: 10.1002/jwmg.596

Pyron RA, O’Connell KA, Lemmon EM, Lemmon AR, Beamer DA. 2020. Phylogenomic data reveal reticulation and incongruence among mitochondrial candidate species in Dusky Salamanders (*Desmognathus*). *Molecular Phylogenetics and Evolution* 146:106751. DOI: [10.1016/j.ympev.2020.106751](https://doi.org/10.1016/j.ympev.2020.106751).

Rainey TA, Tryc EE, Nicholson KE. 2024. Comparing skin swabs, buccal swabs, and toe clips for amphibian genetic sampling, a case study with a small anuran (*Acris blanchardi*). *Biol Methods Protoc*. 16;9:bpae030. DOI: 10.1093/biomethods/bpae030.

Sambrook J, Russell DW. 2001. *Molecular cloning: a laboratory manual*. Cold Spring Harbor, N.Y: Cold Spring Harbor Laboratory Press.

Segev O, Polevikove A, Blank L,Goedbloed D, Küpfer E, Gershberg A, et al. 2015. Effects of Tail Clipping on Larval Performance and Tail Regeneration Rates in the Near Eastern Fire Salamander, *Salamandra infraimmaculata*. *PLoSONE* 10: e0128077. DOI:10.1371/journal.pone.0128077
